# The Early Life History of the Clam *Macoma balthica* in a High CO_2_ World

**DOI:** 10.1371/journal.pone.0044655

**Published:** 2012-09-10

**Authors:** Carl Van Colen, Elisabeth Debusschere, Ulrike Braeckman, Dirk Van Gansbeke, Magda Vincx

**Affiliations:** Ghent University, Department of Biology, Marine Biology Section, Ghent, Belgium; VIB & Katholieke Universiteit Leuven, Belgium

## Abstract

This study investigated the effects of experimentally manipulated seawater carbonate chemistry on several early life history processes of the Baltic tellin (*Macoma balthica*), a widely distributed bivalve that plays a critical role in the functioning of many coastal habitats. We demonstrate that ocean acidification significantly depresses fertilization, embryogenesis, larval development and survival during the pelagic phase. Fertilization and the formation of a D-shaped shell during embryogenesis were severely diminished: successful fertilization was reduced by 11% at a 0.6 pH unit decrease from present (pH 8.1) conditions, while hatching success was depressed by 34 and 87%, respectively at a 0.3 and 0.6 pH unit decrease. Under acidified conditions, larvae were still able to develop a shell during the post-embryonic phase, but higher larval mortality rates indicate that fewer larvae may metamorphose and settle in an acidified ocean. The cumulative impact of decreasing seawater pH on fertilization, embryogenesis and survival to the benthic stage is estimated to reduce the number of competent settlers by 38% for a 0.3 pH unit decrease, and by 89% for a 0.6 pH unit decrease from present conditions. Additionally, slower growth rates and a delayed metamorphosis at a smaller size were indicative for larvae developed under acidified conditions. This may further decline the recruit population size due to a longer subjection to perturbations, such as predation, during the pelagic phase. In general, early life history processes were most severely compromised at ∼pH 7.5, which corresponds to seawater undersaturated with respect to aragonite. Since recent models predict a comparable decrease in pH in coastal waters in the near future, this study indicates that future populations of *Macoma balthica* are likely to decline as a consequence of ongoing ocean acidification.

## Introduction

About one third of the total anthropogenic CO_2_ emissions has currently been absorbed by the oceans, driven by the difference of pCO_2_ in the atmosphere and the surface ocean layers [1]. This results in a continuous increase in seawater pCO_2_ (i.e. hypercapnia) and a decrease in seawater pH and carbonate ion (CO_3_^2−^) availability [2], in a process referred to as ocean acidification. The pH of seawater has already declined by 0.1 unit compared to pre-industrial values and several climate change models predict further decreases up to 0.35 units by the end of this century [3], [4]. However, recent studies indicate that the pH in temperate coastal systems is likely to decrease and order of magnitude faster due to an altered balance between primary production and respiration [5], [6]. These fast-occurring changes may pose far-reaching consequences for marine ecosystems since empirical evidence demonstrates significant alterations in trophodynamics, nutrient cycling, organism physiology, organism reproduction and development as a consequence of ocean acidification (reviewed in e.g. [7]). The implications of ocean acidification for ecosystem resilience are, however, still debated (see e.g. [8]–[10]) because the observed responses are variable and it remains unclear how acidification will interact with other stressors, such as temperature rise, eutrophication and deoxygenation of the oceans.

Meta-analyses and literature reviews suggest that particularly calcification processes are hampered by ocean acidification, e.g. [4], [8], [10], [11]. Subsequently, calcifying organisms are considered specifically susceptible to ocean acidification. The formation of calcium carbonate structures mainly depend on the availability of carbonate ions, given the fact that (1) Ca^2+^ concentrations are rather constant at a given salinity, and (2) the CaCO_3_ saturation state (Ω) is defined as:
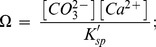
where K’*_sp_* is the stoichiometric solubility product which depends on temperature, salinity, pressure and the mineral phase considered (e.g. high-magnesian calcite, calcite, aragonite). In an acidified ocean, fewer CO_3_^2−^ ions become available to form CaCO_3_ structures and reduced calcification rates have been observed in a variety of calcifying organisms (see e.g. [12] for a review). However, calcification responses to ocean acidification may vary amongst organisms and between life stages, depending on species-specific life-history traits. For example, some organisms may be able to compensate for alteration in carbonate chemistry by increasing calcification rates [13], and organisms which have sufficient energy reserves may be more resilient to ocean acidification [14]. In general, early life history stages are believed to be particularly vulnerable to decreases in seawater pH because CaCO_3_ mineral phases of early life stages (e.g. amorphous CaCO_3_ and aragonite) are often less stable and more soluble than the CaCO_3_ mineral phases which are predominantly precipitated by adults (e.g. calcite) [15]–[17]. Consequently, most ocean acidification studies on the early development of a variety of invertebrate phyla reveal inhibitive effects on larval fitness along with decreasing seawater pH: decreased growth, increased mortality and malformations, e.g. [18]–[24].

Understanding ocean acidification effects on early life history processes is crucial in assessing future ecosystem structure and functioning because settlement and recruitment are fundamental processes in driving ecological balance of communities. This study investigated whether ocean acidification affects several early developmental processes of the Baltic tellin (*Macoma balthica*), from fertilization until metamorphosis. *Macoma balthica* is known to influence sediment nutrient cycling [25], [26] and sediment erodibility in coastal habitats [27]. Furthermore *M. balthica* is a key species in ecosystem carbon flow, because it is a dominant grazer of microalgae and a favourable food source for wading birds, epibenthic crustaceans and fishes [28]–[30]. This species occurs from the Gironde estuary in Southwest France to the polar region in Greenland and Siberia and on the east and west coasts of North America (www.iobis.org), where it often constitutes significant amounts of the benthic metazoan biomass, e.g. [31], [32]. Given the wide distribution of the species and its importance to ecosystem processes, the present study provides a significant contribution to the assessment of future ecosystem functioning in the light of ongoing acidification in coastal and estuarine waters.

## Materials and Methods

### Production of Eggs, Embryos and Larvae: General Procedures

About 600 adult individuals were collected during low tide in April 2011 at the Paulinaschor, a tidal flat located in the polyhaline part of the Westerschelde estuary (51°21′24″N, 3°42′51″E; permits for the field work were approved and obtained by Provincie Zeeland, the Netherlands; Directie Ruimte, Milieu en Water). Test organisms were stored in the lab in containers (40×33×14 cm; about 150 indivuals per container) filled with sieved sediment (1 mm) and aerated 25 PSU filtered seawater at 5°C. They were fed three times a week on a mixed diet of *Isochrysis*, *Pavlova*, *Thalassiosira weissflogii* and *Tetraselmis* (Shellfish Diet 1800; Reed Mariculture). Individual organisms were induced to spawn in 25 PSU 0.2 µm filtered seawater by exposure to the selective serotonin re-uptake inhibitor fluoxetine, preceded by a Δ 10°C temperature shock [33]. On average, 22.3% of the adults could be induced to spawn. Fertilization was carried out by pipetting eggs of several females into a clean glass beaker and adding 1 to 3 ml of a homogenized sperm suspension derived from several males. The resultant mixture was left undisturbed for 4 h at 15°C. Embryos (diameter ∼100 μm) were then separated from all other matter by rinsing them over stacked sieves of 125 and 32 μm. Subsequently, they were transferred into 2.3 L culture flasks containing 15°C UV-irradiated filtered seawater with a salinity of 25 and dosed with 1.5×10^−5^ g.L^–1^ Penicillin G potassium salt and 2.5×10^–5^ g.L^−1^ streptomycin sulphate (further referred to as FSW-AB). The culture flasks were placed on a roller-table (3 rpm) to avoid sinking of embryos and larvae.

**Table 1:** Analysis of variance on difference in early life history processes of *Macoma balthica* reared in the different pH treatments, and correlation with seawater pH.

Developmental parameter	Test statistic	p-level	Post-hoc tests	Correlation with pH
Fertilization success	F = 9.581	0.003	control ≠ ∼pH_7.5_; ∼pH_7.8_ ≠ ∼pH_7.5_	r = 0.71, p = 0.003**
Hatching success	H = 7.200	0.027	control ≠ ∼pH_7.8_ ≠ ∼pH_7.5_	r = 0.97, p<0.001*
Shell size at day 3	H = 7.200	0.027	control ≠ ∼pH_7.8_ ≠ ∼pH_7.5_	r = 0.94, p<0.001*
Growth rate	H = 7.200	0.027	control ≠ ∼pH_7.8_ ≠ ∼pH_7.5_	r = 0.93, p<0.001**
Mortality rate	H = 6.489	0.039	control ≠ ∼pH_7.5_	r = −0.82, p = 0.007*
% of larvae metamorphosed at day 19	H = 1.689	0.430		r = −0.01, p = 0.840*

* indicate results from product moment correlations of logarithmic transformed data,

** indicate results from non-parametric Spearman-rank correlation. For more details: see text.

**Figure 1:**
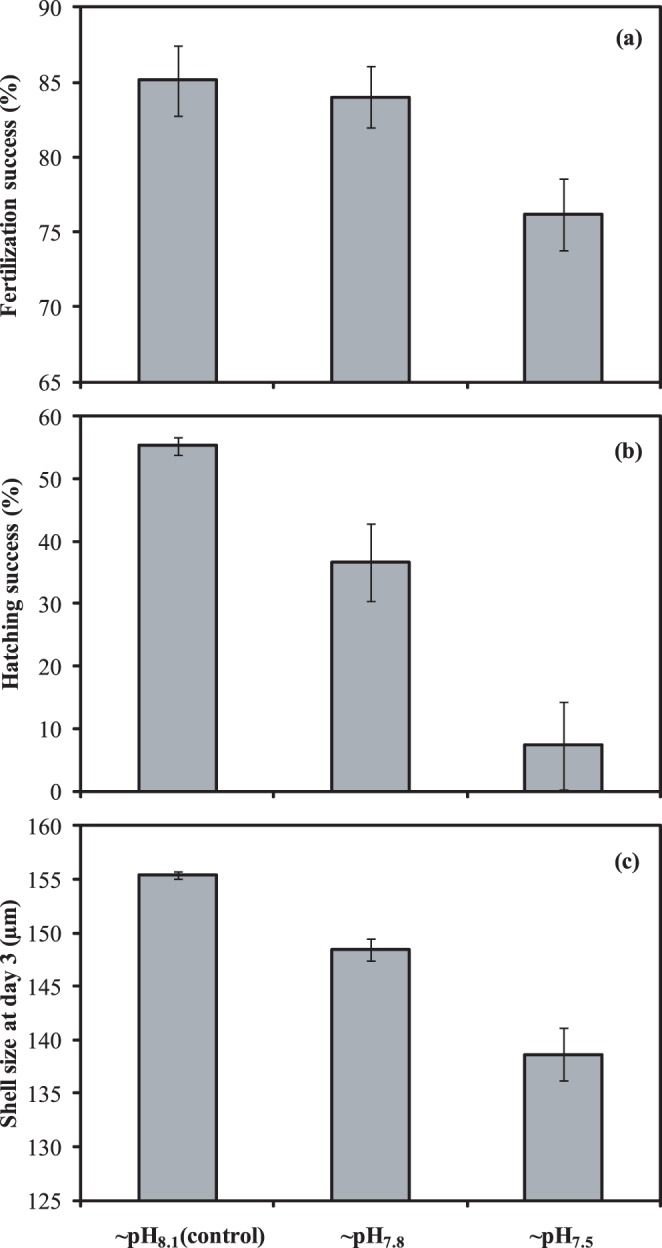
*Macoma balthica*: Ocean acidification effects on fertilization and embryogenesis. (a) fertilization success, (b) hatching success, and (c) size of larvae that have developed a shell after 3 days, reared at three pH conditions: control (∼pH_8.1_), ∼pH_7.8_ and ∼pH_7.5_. Level of replication: n = 5 for fertilization success, n = 3 for hatching success and size. Presented error bars are standard errors.

**Figure 2:**
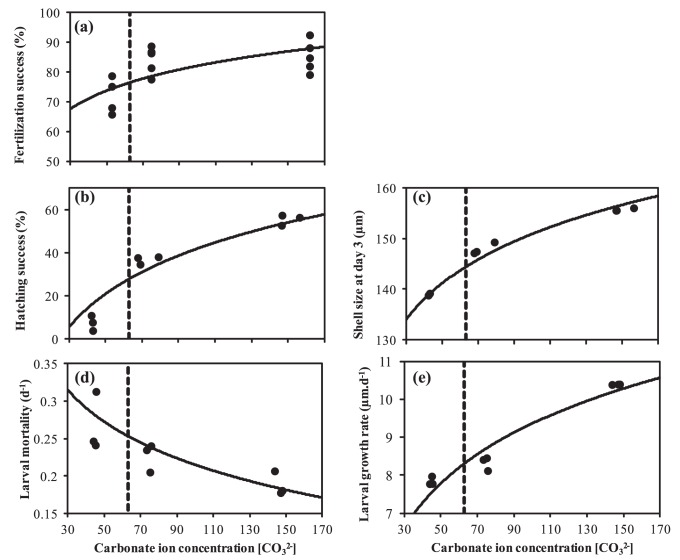
*Macoma balthica*: Relationships between seawater carbonate ion concentration [CO_3_^2−^] and parameters of early life history processes. Fertilization (upper panel; a), embryogenesis (middle panel; b,c), and larval development (lower panel; d,e). The dashed lines refer to the carbonate concentration at the aragonite saturation level (Ω_aragonite_ = 1). The fitted lines show the relationship between the CO_3_^2^ concentration and the developmental parameter according to a Michaelis-Menten equation, e.g. Hatching success = V_max_[CO_3_^2−^]/(K_m_ + [CO_3_^2−^]); where V_max_ is the maximal hatching success and K_m_ is the CO_3_^2^ concentration were the reaction rate is the half of V_max_. Percentage of variation explained (r^2^) by the fitted lines are 0.45 (fertilization success), 0.90 (size at day 3), 0.86 (hatching success), 0.64 (larval mortality rate), and 0.87 (larval growth rate).

### Carbonate Chemistry Manipulation and Measurements

Filtered seawater was diluted with tap water until 25 PSU, i.e. the average salinity at the site where adult organisms were retrieved, and incubated at 15°C and UV-irradiated for 1 week prior to the use in all experiments. Seawater carbonate chemistry was manipulated through direct bubbling of seawater (∼pH 8.1) with 100% CO_2_ gas using Dulcometer technology (ProMinent®) in order to create predicted pH conditions for likely outcomes within the current century in coastal waters: ∼pH 7.8, and ∼pH 7.5 [6], [34]. This method was chosen as it best replicates ocean acidification by altering dissolved inorganic carbon while keeping total alkalinity constant [35]. The environmental parameters and carbonate chemistry conditions that persisted during each experiment are outlined in Tables S1, S2, and S3.

**Figure 3:**
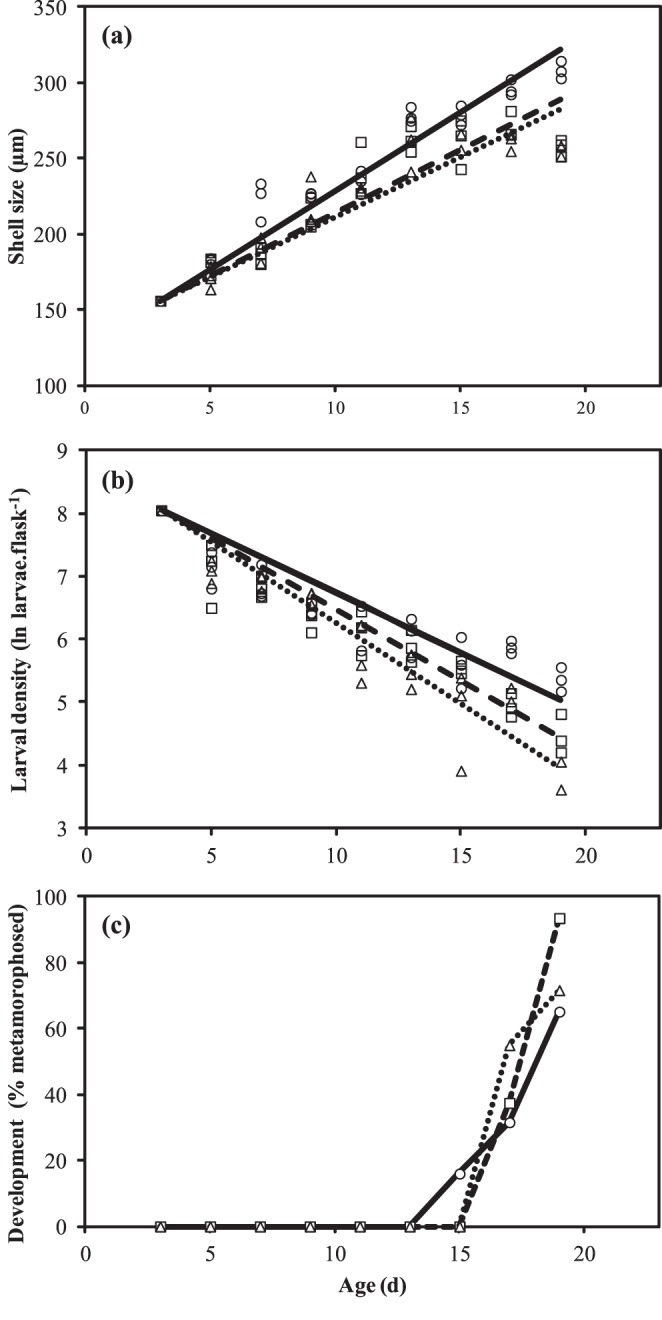
*Macoma balthica*: Ocean acidification effects on larval development. Shell length, survival and development stage of larvae between day 3 and day 19 of development reared at three seawater pH conditions: ∼pH_8.1_ (circles, solid line), ∼pH_7.8_ (squares, dashed line), and ∼pH_7.5_ (triangles, dotted line). Regression lines in (a) and (b) indicate average larval growth rate (µm.d^−1^) and mortality rate (d^−1^) for each treatment.

The carbonate chemistry manipulation unit comprised two incubation tanks (60 L and 15 L) for each pH treatment. Seawater was pumped constantly between the two tanks in order to mix the seawater (5 L.min^−1^) and one third of the total volume was replaced every day by freshly incubated seawater. The 15 L incubation tank was constantly bubbled with ambient air to maintain dissolved oxygen levels >90%. The carbonate chemistry was manipulated through controlled pumping of 100% CO_2_ gas at the bottom of the 60 L incubation tank, using a pH controller (Dulcometer D1C, version B) connected to a pH electrode (Dulcotest PHE-112SE) mounted in the 60 L incubation tank. Every other day the pH electrodes were calibrated using Hanna Instruments’ pH_NBS_ buffers. The control seawater unit had the same set-up except for the CO_2_ gas supply and was bubbled with ambient air. Seawater with the targeted pH was pumped from the manipulation units directly into the culture flasks without leaving headspace to avoid water-atmosphere gas exchange [35]. Temperature and salinity (WTW COND 330), pH (Dulcotest PHE-112SE) and total alkalinity (TA) were measured prior and at the end of each incubation in the different experiments, as further described below. Total alkalinity was determined on 20 ml samples filtered on GF/F membranes by end-point titration of 25 mmol.L^−1^ HCl, using a Mettler Toledo G20 Compact Titrator. The recorded values were entered in CO2SYS software [36] to calculate the remaining carbonate chemistry parameters using the thermodynamic constants of Merhbach et al. [37]: partial CO_2_ pressure, total concentration of dissolved inorganic carbon (DIC), concentration of HCO_3_^−^ and CO_3_^2−^, saturation state of aragonite and calcite. We are aware that the applied method for alkalinity determination differs from the general procedure applied in the ocean acidification community (Gran Titration, [38]); however our measurements of Dickson CRM water (batch 107) had, on average, only 3.35% higher TA values. Since these enhanced TA values resulted in only slightly overestimated concentrations of CO_3_^2−^ (i.e. on average +20.7, +10.6, +6.4 µmol.kg^−1^ for control, ∼pH_7.8_ and ∼pH_7.5_ conditions) and aragonite saturation states (i.e. on average +0.33, +0.17, +0.10 for control, ∼pH_7.8_ and ∼pH_7.5_ conditions) we are confident that the obtained results and conclusions deduced from this study are not compromised.

### Ocean Acidification Effects on Fertilization Success

Fertilization was carried out by pipetting 50 eggs into clean vials containing 23 ml of control, ∼pH_7.8_ and ∼pH_7.5_ seawater. Subsequently, 2 ml of the sperm suspension was added. Five replicate vials of each pH treatment were continuously and gently shaken during incubation at 15.2°C for 24 hours. Embryonic development was stopped by adding 1 ml of a neutralized 4% formaldehyde – tap water solution. Unfertilized eggs and the embryos already developed were counted using a Leica MZ 16 stereomicroscope and the fertilization success was calculated as:
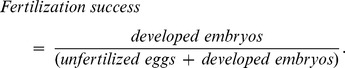



### Ocean Acidification Effects on Embryonic Development

Adults were induced to spawn and eggs were fertilized under control conditions according to the general procedures described above. Subsequently, embryos were equally distributed into triplicated 2.3 L culture flasks (2.2 embryos ml^−1^) containing 15.2°C control, ∼pH_7.8_ and ∼pH_7.5_ FSW-AB and left to incubate on a roller-table. After 3 days, five 4 ml aliquots were collected from each culture flask and embryonic development was stopped by adding 1 ml of a neutralized 4% formaldehyde – tap water solution to the aliquot. The shell length of all embryos that had developed a shell were measured to the nearest 0.1 µm under a stereomicroscope using a camera lucida mounted to a stereomicroscope and Image J 1.44 software. In addition, the density of unfertilized eggs, embryos that have developed to a normal D-shaped shell characterized by a straight hinge, and abnormal developed larvae (i.e. shell with a convex hinge or protruding mantle; [39]) was determined in order to calculate hatching success according to:
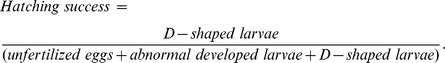



### Ocean Acidification Effects on Larval Development

Adults were induced to spawn and embryos were developed under control conditions according to the general procedures described above. Subsequently, 3 day-old larvae were equally distributed into triplicated 2.3 L culture flasks (1.4 larvae.ml^−1^) containing 14.8°C control, ∼pH_7.8_ and ∼pH_7.5_ FSW-AB and 10^5^ live *Isochrysis galbana* cells.ml^−1^ originating from a continuously maintained healthy culture in the laboratory. The larval cultures were incubated on a roller-table for 16 days (i.e. in total 19 days after fertilization) and refreshed every other day by rinsing the seawater and larvae over a 32 µm mesh-sized sieve and transferring the larvae into new culture flasks containing control, ∼pH_7.8_ and ∼pH_7.5_ FSW-AB and 10^5^ live *I. galbana* cells.ml^−1^. Seawater subsamples (25 ml) were collected from each culture flask at the start and end of incubation in order to calculate the carbonate chemistry parameters. Additionally, three subsamples were taken every two days from each culture flask to measure larval mortality, growth and metamorphosis stage. Following Bos et al. [40], [41] we obtained:

1. mortality rates from fitting a regression line through the natural logarithms of the densities through time, according to:

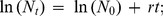
where *N_t_* is the density of larvae at time t (larvae.ml^−1^), *N_0_* the initial density at day 3 (larvae.ml^−1^), *r* is the mortality rate (d^−1^), *t* is the age (d) of the larvae;

1. growth rates from the linear model:

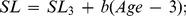
where SL is the shell length (µm), SL_3_ is the initial shell length (µm) at day 3 and *b* is the growth rate (µm.d^−1^);

1. average shell length at metamorphosis from the logistic regression:

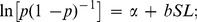
where *p* is the probability of passing metamorphosis (i.e. the presence of a clearly visible foot bulging out of the shell). The age at which 50% of the larvae had metamorphosed (i.e. reaction norm) was calculated by setting *p* at 0.5 in the logistic regression model; yielding the linear predictor, *lp* = 0. The average age at which 50% of the larvae reach metamorphosis was subsequently calculated from the modified equation (2):



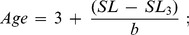
where SL is the shell length at metamorphosis deduced from the logistic regression.

### Data Analysis

One-way analysis of variance, followed by Tukey HSD post-hoc tests, was used to interpret the significance of ocean acidification effects on fertilization success (n = 5), after testing for normality (Shapiro-Wilk’s test) and homoscedasticity (Levene test). Kruskal-Wallis rank sum tests followed by Conover’s multiple comparison tests [42] were applied to investigate ocean acidification effects on hatching success, the percentage of metamorphosed larvae at day 19, larval mortality and growth rates (n = 3). For all tests, differences were considered significant at p<0.05. In order to interpret relationships of developmental parameters with the manipulated seawater carbonate chemistry we performed correlations with pH. In addition, Michaelis-Menten functions were fitted to the CO_3_^2−^ ion concentration since the latter parameter is considered the main governing factor of larval shellfish growth [23].

## Results

### Fertilization and Embryonic Development

Manipulation of seawater carbonate chemistry significantly altered egg fertilization and embryonic development to a D-shaped larvae (Table 1). On average, only 76.19±0.02 SE % of the eggs were successfully fertilized at ∼pH_7.5_, which was respectively 9 and 8% lower as compared to fertilization under control and ∼pH_7.8_ conditions (Fig. 1a). In addition, significantly fewer embryos were able to produce a normal D-shape after 3 days of development at ∼pH_7.8_ and ∼pH_7.5_ as compared to control embryos (Fig. 1b). Hatching success for embryos reared at ∼pH_7.5_ were 48 and 29% lower as compared to embryos reared under control and ∼pH_7.8_ conditions, respectively. On average, 3 day-old control embryos that have developed a shell measured 155.4±0.4 SE µm, which was significantly larger than embryos reared under acidified conditions: i.e. shell size was 148.4±1.0 SE µm and 138.7±2.5 SE µm, respectively at ∼pH_7.8_ and ∼pH_7.5_ (Fig. 1c). Simple correlations indicate that fertilization and embryonic development significantly decreased with decreasing pH (Table 1), while non-linear regressions indicate that decreasing CO_3_^2−^ concentrations resulted in smaller larvae and a drastically lower hatching success, especially below the aragonite saturation level (Ω_aragonite_) (Fig. 2b, c).

### Larval Development

Larval development was significantly affected by the carbonate chemistry of the seawater (Table 1). The initial shell length was 156.0±1.0 SE µm at day 3 for all treatments. At day 19, larvae grown under control conditions had an average shell length of 308.4±3.4 SE µm, while larvae grown under acidified conditions had an average shell length of 257.6±3.2 SE µm (∼pH_7.8_) and 256.1±2.3 SE µm (∼pH_7.5_). The initial density of larvae was 1.36 larvae.ml^−1^ at day 3. Under control conditions, the larval density was reduced by 93% to 0.10±0.01 SE larvae.ml^−1^ at day 19. Mortality of larvae that developed under acidified conditions was even more pronounced (97±0.007 SE % and 99±0.004 SE %, respectively at ∼pH_7.8_ and ∼pH_7.5_). Thus, larvae grow more slowly and suffer greater mortality under acidified conditions (Fig. 3a, b), as was confirmed by the decrease in growth rate and increase in mortality rates when fewer CO_3_^2−^ ions became available (Table 1) (Fig. 2d, e). However, multiple comparison tests only detected significantly different mortality rates between larvae that developed under control conditions and at ∼pH_7.5_.

There was no significant difference among treatments in the percentage of larvae that had metamorphosed at day 19 (Fig. 3c). However logistic regression indicated that, on average, larvae reared under acidified conditions metamorphosed at a smaller size (267.05 µm and 261.35 µm at ∼pH_7.8_ and ∼pH_7.5_, respectively) which was reached later in the development process, i.e. after 16.3–16.4 days, as compared to larvae that have developed under control conditions (size at metamorphosis = 281.5 µm, age at metamorphosis = 15.1 days).

## Discussion

This study illustrates that ocean acidification may negatively affect shellfish recruitment success by impacting multiple early life history processes prior to settlement, including egg fertilization, embryonic shell formation (i.e. hatching success), larval mortality, growth and metamorphosis.

Results of the fertilization experiment demonstrate that failure of fertilizations increase with enhanced seawater pCO_2_. Similar effects have been found for a variety of invertebrate phyla and have been attributed to a reduction in the efficiency to block polyspermy and a reduction in sperm speed and motility which decreases fertilization success (e.g. [43]–[46]). In addition, the intracellular egg pH has been shown instrumental to successful fertilization of sea urchin eggs by regulating sperm entrance through the egg membrane [47]. This mechanism may therefore be distorted when more CO_2_ diffuses across the gamete cell membrane in an acidified sea. So far studies of ocean acidification effects on bivalve fertilization have yielded variable results. This suggests that effects may be species-specific and reflect the adaptation of the species to the pH variability in the species’ habitat, and that effects may be influenced by site-specific environmental parameters, such as temperature and eutrophication. For example, Parker et al. [48] demonstrated that fertilization in a population of the Pacific oyster *Crassostrea gigas* from Port Stevens (Australia) was significantly reduced at elevated CO_2_, while Havenhand & Schlegel [49] did not detect a significant effect for a *C. gigas* population from Western Sweden. We found a significant reduction in fertilization success of *Macoma balthica* at ∼pH_7.5_ but not at ∼pH_7.8_ which may indicate adaptation to fluctuating pH conditions that characterize the habitat of the studied population, i.e. pH 8.2–7.8 [50].

Under acidified conditions, *Macoma balthica* embryos were still able to form a shell, even in seawater undersaturated with respect to aragonite. However, larval growth rates were significantly reduced under acidified conditions, which corroborate other research on bivalve early life history development, e.g. [12], [18], [23], [24], [51]. Non-linear relationships between seawater CO_3_^2−^ concentrations and growth rate and hatching success, with a sharp decrease below the aragonite saturation level, demonstrate the importance of the amount of available CO_3_^2−^ ions to form CaCO_3_ shells. The observed higher larval mortality and a longer development time of larvae in acidified seawater likely reflects a higher energy demand to form a shell when less CO_3_^2−^ ions are available.

Recruitment, which demands successful larval settlement and post-settlement growth and survival is critical to the persistence and resilience of benthic populations. Pre-recruitment mortality of planktonic invertebrate larvae is variable but generally accepted to be very high (3–23% daily), particularly due to predation and environmental factors [52], [53]. Ocean acidification thus exerts a significant additional burden to the recruitment of *Macoma balthica* by negatively impacting multiple early life history processes. Assuming that abnormally developed 3 day-old larvae will not develop to the benthic stage, the quantified effects of decreasing seawater pH on fertilization, embryogenesis and larval development in this study suggest that the number of *M. balthica* larvae that are competent to settle will decline up to 38–89%, depending on the scenario (i.e. respectively for a 0.3 and 0.6 pH unit decrease from present conditions). Additionally, a delayed metamorphosis at a smaller larval size may further diminish the recruit population size due to a longer subjection to perturbations (e.g. predation) during the pelagic phase. As *Macoma balthica* is recognized as an ecosystem engineer to coastal habitats, e.g. [26], [54], ocean acidification-mediated changes to this species’ population size and distribution may affect coastal diversity and ecosystem functioning.

## Supporting Information

Table S1 Environmental parameters and carbonate chemistry for the different treatments to investigate ocean acidification effects on fertilization success. Carbonate parameters were computed from the recorded seawater pH_NBS_ and Total Alkalinity during the incubations. - : no data available.
(XLSX)

Table S2 Environmental parameters and carbonate chemistry for the different treatments to investigate ocean acidification effects on embryonic development. Carbonate parameters were computed from the recorded seawater pH_NBS_ and Total Alkalinity during the incubations.
(XLSX)

Table S3 Environmental parameters and carbonate chemistry for the different treatments to investigate ocean acidification effects on larval development. Carbonate parameters were computed from the recorded seawater pH_NBS_ and Total Alkalinity during the incubations. - : no data available.
(XLSX)
